# Supplementary material for: Novel Gene Signatures as Prognostic Biomarkers for Predicting the Recurrence of Hepatocellular Carcinoma
Source: Cancers (Basel). 2022 Feb 9;14(4):865. doi: 10.3390/cancers14040865 (PMC8870597; doi:10.3390/cancers14040865)
Supplement: Supplementary file 1 [file cancers-14-00865-s001.zip › Supplement Table S1.pdf]

Supplementary Table S1. Baseline characteristics of 57 patients with HCC in validation set

Variables	Total patients (n=57)
**Male sex, n (%)**	44 (77.2)
**Age (years), mean±SD**	55.8±10.2
**Liver cirrhosis, n (%)**	22(40)
**Platelet (10^9/L), mean±SD**	180.246±67.532
**Albumin (g/dL), mean±SD**	4.453±0.759
**Total bilirubin (mg/dL), mean±SD**	0.877±1.164
**Creatinine (mg/dL), mean±SD**	0.857±0.198
**Serum AST (U/L), mean±SD**	55.912±87.949
**Serum ALT (U/L), mean±SD**	48.912±61.177
**Serum AFP (ng/mL), mean±SD**	3596.948±11154.173
**PIVKA II (mAU/ml), mean±SD**	11544.551±43573.442
**Micro Vascular invasion, n(%)**	18(31.579)
**BCLC stage, n(%) 0/A/B/C**	12(21.053)/26(45.614)/2(3.509)/17(29.825)
**Modified UICC stage, n (%) I/II/III/IVA/IVB**	14(24.561)/23(40.351)/15(26.316)/2(3.509)/3(5.263)
**Edmondsongrade, n(%) 1/2/3/4**	0(0)/5(9.091)/24(43.636)/26(47.273)
**Recurrence, n(%)**	20(35.088)
**OS_month, mean±SD, n**	17.386±10.359, 57
**DFS_month, mean±SD, n**	13.211±10.574, 54

AST, aspartate aminotransferase; ALT, alanine aminotransferase; AFP, α-fetoprotein; PIVKA II, Protein induced by vitamin K absence-II; OS, overall survival; DFS, disease-free survival
