# Supplementary material for: Novel Gene Signatures as Prognostic Biomarkers for Predicting the Recurrence of Hepatocellular Carcinoma
Source: Cancers (Basel). 2022 Feb 9;14(4):865. doi: 10.3390/cancers14040865 (PMC8870597; doi:10.3390/cancers14040865)
Supplement: Supplementary file 1 [file cancers-14-00865-s001.zip › Supplement Table S2.pdf]

Supplement Table S2. List of primers sequences

**Gene**	Accession No.		**Nucleotide Sequence**
CETN2	NM_004344.3	Forward	5'-TGTACACGTCGGTTGCCTAA-3'
		Reverse	5'-TTCCCGGATCTCCTGCTTTT-3'
HMGA1	NM_145901.3	Forward	5'-ACCAGCGCCAAATGTTCATCCTCA-3'
		Reverse	5'-AGCCCCTCTTCCCCACAAAGAGT-3'
MPZL1	NM_003953.6	Forward	5'-ACGCCAAAAGAAATCTTCGTGG-3'
		Reverse	5'-TCAACCCGCCAGTCGTACTA-3'
RACGAP1	NM_013277.5	Forward	5'-ACGTTGAATAGGATGAGTCATGGA-3'
		Reverse	5'-AAAGTCCTTCGCCAACTGGA-3'
SNRPB	NM_003091.4	Forward	5'-CCTGGTATGAGACCTCCTAGTG-3'
		Reverse	5'-TGCGGAGCTACTTCCATACTCT-3'
HMBS	NM_001024382.2	Forward	5'-GGAGGGCAGAAGGAAGAAAACAG-3'
		Reverse	5'-CACTGTCCGTCTGTATGCGAG-3'
