# Supplementary material for: Novel Gene Signatures as Prognostic Biomarkers for Predicting the Recurrence of Hepatocellular Carcinoma
Source: Cancers (Basel). 2022 Feb 9;14(4):865. doi: 10.3390/cancers14040865 (PMC8870597; doi:10.3390/cancers14040865)
Supplement: Supplementary file 1 [file cancers-14-00865-s001.zip › Supplement Table S3.pdf]

Supplementary Table S3. Enriched gene set correlated with recurrence by GSEA

Gene set	No.	GENE SYMBOL	RUNNING ES
WOO_LIVER_CANCER_RECURRENCE_UP	1	HMGA1	0.1209
	2	LAMA5	0.2387
	3	ALDOA	0.4414
	4	ARHGEF2	0.4913
	5	MYO6	0.6019
	6	E2F3	0.5201
	7	TSPAN3	0.6214
YOSHIOKA_LIVER_CANCER_EARLY_ RECURRENCE_UP	8	DFNA5	0.0845
	9	SNRPB	0.206
	10	NARF	0.2207
	11	LCMT1	0.3525
	12	DNAJC8	0..4227
WANG_RECURRENT_LIVER_CANCER_UP	13	RACGAP1	0.1926
	14	CETN2	0.3391
	15	SH3GLB2	0.374
	16	CUL4B	0.5645
HOSHIDA_LIVER_CANCER_LATE_RECURRENCE_UP	17	MPZL1	0.1518
	18	RALY	0.3036
	19	RNF26	0.4082
