# Supplementary material for: Novel Gene Signatures as Prognostic Biomarkers for Predicting the Recurrence of Hepatocellular Carcinoma
Source: Cancers (Basel). 2022 Feb 9;14(4):865. doi: 10.3390/cancers14040865 (PMC8870597; doi:10.3390/cancers14040865)
Supplement: Supplementary file 1 [file cancers-14-00865-s001.zip › Supplement Table S4.pdf]

Supplementary Table S4. AUCs of AFP and 5 core genes

Single marker			
	AUC	95% CI	P value
AFP	0.628	0.490 to 0.752	—
CETN2	0.689	0.553 to 0.805	0.5176
HMGA1	0.755	0.623 to 0.859	0.1764
MPZL1	0.524	0.387 to 0.658	0.4023
RACGAP1	0.754	0.622 to 0.858	0.1873
SNRPB	0.677	0.540 to 0.795	0.6255
