# Supplementary material for: Novel Gene Signatures as Prognostic Biomarkers for Predicting the Recurrence of Hepatocellular Carcinoma
Source: Cancers (Basel). 2022 Feb 9;14(4):865. doi: 10.3390/cancers14040865 (PMC8870597; doi:10.3390/cancers14040865)
Supplement: Supplementary file 1 [file cancers-14-00865-s001.zip › Supplement Table S5.pdf]

Supplementary Table S5. AUCs of the combination of AFP and 5 core genes

Combination marker with AFP			
	AUC	95% CI	P value
AFP	0.628	0.490 to 0.752	—
AFP + CETN2	0.696	0.560 to 0.811	0.4173
AFP + HMGA1	0.755	0.624 to 0.860	0.1341
AFP + MPZL1	0.597	0.459 to 0.725	0.6280
AFP + RACGAP1	0.749	0.616 to 0.854	0.1933
AFP + SNRPB	0.716	0.581 to 0.828	0.3009
