# Supplementary material for: Novel Gene Signatures as Prognostic Biomarkers for Predicting the Recurrence of Hepatocellular Carcinoma
Source: Cancers (Basel). 2022 Feb 9;14(4):865. doi: 10.3390/cancers14040865 (PMC8870597; doi:10.3390/cancers14040865)
Supplement: Supplementary file 1 [file cancers-14-00865-s001.zip › Supplement Table S6.pdf]

Supplementary Table S6. AUCs of the combination of two gene signatures

Duo marker			
	AUC	95% CI	P value
AFP	0.628	0.490 to 0.752	—
CETN2 + HMGA1	0.795	0.667 to 0.890	0.0501
HMGA1 + MPZL1	0.807	0.681 to 0.899	0.0410
HMGA1 + RACGAP1	0.780	0.650 to 0.879	0.0995
MPZL1 + RACGAP1	0.812	0.687 to 0.903	0.0551
SNRPB + HMGA1	0.761	0.629 to 0.864	0.1582
